# Structural effects of naphthalimide-based fluorescent sensor for hydrogen sulfide and imaging in live zebrafish

**DOI:** 10.1038/srep26203

**Published:** 2016-05-18

**Authors:** Seon-Ae Choi, Chul Soon Park, Oh Seok Kwon, Hoi-Khoanh Giong, Jeong-Soo Lee, Tai Hwan Ha, Chang-Soo Lee

**Affiliations:** 1 BioNanotechnology Research Center, Korea Research Institute of Bioscience and Biotechnology (KRIBB), 125 Gwahak-ro Yuseong-gu, Daejeon 305-806, South Korea; 2 Nanobiotechnology (Major), University of Science & Technology (UST), 125 Gwahak-ro Yuseong-gu, Daejeon 305-806, South Korea; 3 Functional Genomics (Major), University of Science & Technology (UST), 125 Gwahak-ro Yuseong-gu, Daejeon 305-806, South Korea

## Abstract

Hydrogen sulfide (H_2_S) is an important biological messenger, but few biologically-compatible methods are available for its detection in aqueous solution. Herein, we report a highly water-soluble naphthalimide-based fluorescent probe (**L**_**1**_), which is a highly versatile building unit that absorbs and emits at long wavelengths and is selective for hydrogen sulfide over cysteine, glutathione, and other reactive sulfur, nitrogen, and oxygen species in aqueous solution. We describe turn-on fluorescent probes based on azide group reduction on the fluorogenic ‘naphthalene’ moiety to fluorescent amines and intracellular hydrogen sulfide detection without the use of an organic solvent. **L**_**1**_ and **L**_**2**_ were synthetically modified to functional groups with comparable solubility on the N-imide site, showing a marked change in turn-on fluorescent intensity in response to hydrogen sulfide in both PBS buffer and living cells. The probes were readily employed to assess intracellular hydrogen sulfide level changes by imaging endogenous hydrogen sulfide signal in RAW264.7 cells incubated with **L**_**1**_ and **L**_**2**_. Expanding the use of **L**_**1**_ to complex and heterogeneous biological settings, we successfully visualized hydrogen sulfide detection in the yolk, brain and spinal cord of living zebrafish embryos, thereby providing a powerful approach for live imaging for investigating chemical signaling in complex multicellular systems.

Hydrogen sulfide (H_2_S), an endogenously produced gaseous signaling compound and important biological messenger, has recently been recognized as a gasotransmitter with two other known endogenous gasotransmitters, nitric oxide (NO) and carbon monoxide (CO)^1^,^2^. The production of endogenous hydrogen sulfide and the exogenous administration of hydrogen sulfide have been verified to exert protective effects in many pathologies, such as relaxing vascular smooth muscle, inducing vasodilation of isolated blood vessels, and reducing blood pressure^1^,^2^,^3^. The endogenous levels of hydrogen sulfide in the cell are tightly controlled, and it is produced, as a by-product in three enzyme pathways by cystathionine-*γ*-lyase (CSE), cystathionine-*β*-synthase (CBS), and 3-mercapto-sulfurtransferase (MST)^4^,^5^,^6^,^7^.

Furthermore, the concentration of hydrogen sulfide has been proven to have close relation to particular diseases; for example, it is excessively produced in sepsis and is found at very low levels in Down’s syndrome^8^ and Alzheimer’s disease^9^. Although the hydrogen sulfide level in biological systems is known to be related to numerous physiological and pathological processes, many underlying molecular events remain undefined. Moreover, because the sulfide concentration in blood is in the range of 10–100 μM^10^,^11^,^12^,^13^,^14^ or lower^15^,^16^, new effective methods for highly sensitive hydrogen sulfide detection in living biological systems are needed. At present, hydrogen sulfide detection is mainly performed through colorimetric and electrochemical assays, gas chromatography, and sulfide precipitation^17^,^18^,^19^,^20^,^21^, which often require complex sample processing.

Moreover, the catabolism of hydrogen sulfide is quick, which can result in continuous changes in its concentration, leading to difficulty in the accurate analysis^22^. However, existing detection methods have limitations in terms of their response rate, accuracy, and lack of real-time determination; the most important factor in sensing hydrogen sulfide is the lack of sensors and agents that allow for its rapid and accurate detection. Among recently developed biological detection technologies of hydrogen sulfide, fluorescence-based methods provide greater selectivity, more convenience, less invasiveness, and high sensitivity *in situ* as well as in real-time imaging^23^,^24^,^25^,^26^,^27^,^28^. A variety of fluorescent probes have been designed on the basis of the reactions of hydrogen sulfide to detect hydrogen sulfide in solutions and cells by reducing azido or nitro groups on the fluorogenic moiety, such as rhodamine, fluorescein and cyanine^29^,^30^,^31^. Taking advantage of the known unique reduction of an azido group by hydrogen sulfide can be useful in developing a sulfide-sensitive agent^32^. Moreover, the strongly electron-withdrawing group of naphthalimide accelerates the reduction of an azido group^33^. 1,8-Naphthalimide is a cell-permeable fluorophore with a visible emission wavelength and high photostability. In general, substituted naphthalimide show strong intramolecular charge transfer (ICT) in the solution state arising from their planar architecture combined with the electron-withdrawing ability of the imide core. However, this naphthalimide-based fluorescent reporter has many undesirable properties such as low water solubility, furthermore, minor changes in the environment such as temperature and oxygen concentration^34^. Therefore, in making such hydrogen sulfide sensors using the naphthalimide-based fluorophore, it is always necessary to add some organic co-solvent, particularly, for the living cell studies. Synthesis of various fluorescent probes can be accomplished easily by introducing different functional groups to the aromatic naphthalene moiety and ‘N-imide site’. Herein, we report the use of a naphthalimide-based structure as an important class of organic fluorophores, which has a unique photophysical properties and has recently been applied to many areas of chemical and biological sensing^35^,^36^,^37^,^38^, and to the determination of hydrogen sulfide in aqueous solution. The various photophysical properties of the naphthalimide structure can be easily tuned through suitable structural design, such as a functionalization to the aromatic naphthalene moiety and ‘N-imide site’, showing absorption and fluorescence emission spectra within the UV and visible regions. Naphthalimide has also been used within the dye industry, in strongly absorbing and colorful dyes, in the construction of novel therapeutics^39^, and in the formation of chemiluminescent probes^37^,^40^,^41^,^42^, especially for the detection of biologically relevant cations^36^,^37^,^38^. In this study, we synthesized two fluorescent probes, **L**_**1**_ and **L**_**2**_, as shown in Fig. 1, expecting different characteristics to depend on the substituted chains at the ‘N-imide site’ of the naphthalimide structure. The introduction of distinct alkyl chain has a notable effect on its solubility in aqueous media, consequently, the capability to respond to sulfide sources, such as fluorescence intensity, selectivity for various analytes, cell permeability and live animal imaging^43^. Therefore, our highly water-soluble probes for hydrogen sulfide are appealing, owing to their greater ability for quantitative tracking compared with ratiometric hydrogen sulfide probes that have previously been reported. **L**_**1**_ itself is non-fluorescent; however, it showed a strong fluorescence enhancement upon the addition of hydrogen sulfide. The current work describes the synthesis of a highly water-soluble fluorescent probe **L**_**1**_ for selective hydrogen sulfide detection, comparing the optical and biological properties, such as fluorescent intensity and cytotoxicity in living cells along with the relatively lower solubility of **L**_**2**_. Finally, we report the visualization of bright fluorescent signal through the exogenous-responsive hydrogen sulfide detection in live zebrafish.

## Results and Discussion

Hydrogen sulfide participates in nucleophilic substitution as a reactive nucleophile in biological systems. A number of hydrogen sulfide probes based on the reduction of aromatic azide show a delayed response time (>20 min) toward hydrogen sulfide^24^,^44^. To improve the reaction rate, an electron-withdrawing group, fluorine, on the o-position of the aromatic azide can be introduced^45^. Along with the consideration of the physiological properties of aromatic azide group, the introduced functional group on the ‘N-imide site’ of our probes affected properties such as the fluorescent intensity, response time and cell permeability, as well as the solubility in aqueous solution. The synthetic procedure for both probes **L**_**1**_ and **L**_**2**_ is outlined in Fig. S1, and the NMR and mass data for all products are also shown in Figs S2 and S5. Whereas azide derivatives typically display low fluorescence intensity, the on-off fluorescence response is obtained after reduction to the amine counterpart fluorescence, which is strongly based on the thiolate-triggered reaction in the presence of hydrogen sulfide^46^,^47^.

**Figure 2:**
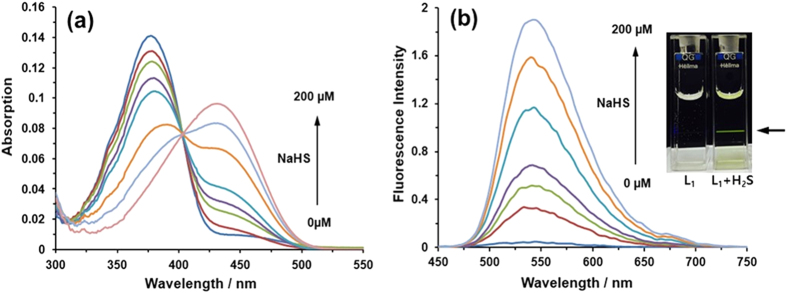
(**a**) Time-dependent absorption spectra of **L**_**1**_ (10 μM) with NaHS (0, 10, 20, 30, 50, 100, 150 and 200 μM) in PBS buffer (pH 7.4) at 37 °C for 30 min. (**b**) Time-dependent fluorescence spectra of **L**_**1**_ (10 μM) with NaHS (0, 10, 20, 30, 50, 100, 150 and 200 μM) in PBS (pH 7.4) at 37 °C for 30 min. The resulting bright green fluorescent enhancement (inset).

**Figure 3:**
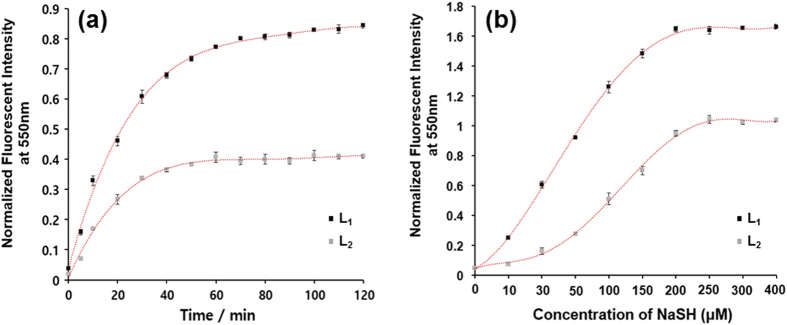
(**a**) Reaction time profile of **L**_**1**_ (10 μM) and **L**_**2**_ (10 μM) with NaHS (100 μM) in PBS (pH 7.4) buffer at 37 °C, (**b**) Fluorescence spectra of **L**_**1**_ (10 μM) and **L**_**2**_ (10 μM) with NaHS (0, 10, 30, 50, 100, 150, 200, 250, 300 and 400 μM) in PBS buffer (pH 7.4) at 37 °C for 30 min.

We investigated the absorbance spectra of **L**_**1**_ and its reaction with hydrogen sulfide using NaHS (a common hydrogen sulfide source) in PBS buffer (10 μM, pH 7.4) at 37 °C. All experiments for **L**_**1**_ were conducted without the use of DMSO as a co-solvent, because **L**_**1**_ displays remarkable solubility in aqueous buffer solution. Naphthalimide-based structure itself is essentially non- or low fluorescent in aqueous solution. As shown in Fig. 2a, the UV-vis spectra of **L**_**1**_ exhibited two noticeable absorption bands at approximately 350–400 nm and 400–500 nm. The probe exhibited absorbance originating from the naphthalene moiety at 350–400 nm, as followed by an obvious increase of new absorbance peak at 435 nm after treatment with hydrogen sulfide. The large red shift of 60 nm in the absorption behavior induced a color change of the solution from colorless to yellow (Fig. S6), thus allowing the colorimetric detection of hydrogen sulfide by the naked eye. The comparable color change for both **L**_**1**_ and **L**_**2**_ upon titration with hydrogen sulfide was distinguished, depending on the structure of probes. As predicted, **L**_**1**_ exhibited a high quantum yield (Φ_**L1**_ = 0.62) in aqueous media when excited at the λ_max_ (457 nm) of **L**_**1**_. The consequential bright green fluorescent enhancement was also observed by 457 nm laser irradiation along with the increased absorbance (Fig. 2b inset). Accordingly, the titration of probes with hydrogen sulfide was performed, and emission at 550 nm clearly appeared upon excitation at 435 nm (Fig. 2b), which reflected that the azide group of **L**_**1**_-N_3_ was converted by efficient reduction into fluorescent **L**_**1**_-NH_2_. The fluorescent signal increase produced by an approximately 70-fold turn-on response, when the ratio of emission intensities (*I*_*550*_ _*nm*_*/I*_*435*_ _*nm*_) varied from 0.028 to 1.9, was observed over 30 min of reaction time without any background correction (Fig. 2b). The electronic spectra of **L**_**1**_ and **L**_**2**_ were recorded in PBS buffer at pH 7.4. Comparing of **L**_**1**_ and **L**_**2**_, a relatively higher absorbance and fluorescence intensity for **L**_**1**_ was obvious (Fig. S7); this was expected, given that the introduced chemical structure group in the side chain on the ‘N-imide site’ led to the enhancement of the fluorescence intensity. **L**_**1**_ and **L**_**2**_ differ significantly in their molecular structures, therefore, one can envision different degrees of intermolecular interactions in solution phases. Incorporated hydroxy substituents enhanced the water solubility and reduced the potential for aggregation. Additionally, the abundant oxygen groups influenced on the enhanced solubility. In addition to this enhancement, the fluorescent emission maxima varied in the range of λ = 540–550 nm. The electronic effect of introducing a hydrophilic structure is ambiguous; however, this structural changes might prevent aggregation effects^48^. The linear relationship suggests that **L**_**1**_ and **L**_**2**_ can be used to determine reaction time- and concentration-dependent fluorescence responses for hydrogen sulfide by measuring the fluorescence at 550 nm. Because the linear relationship is significant for accurate analysis, the dependence of fluorescence changes on the hydrogen sulfide concentration and response time was examined quantitatively, including in aqueous solution. The time-dependent fluorescence responses of **L**_**1**_ and **L**_**2**_ were detected with the addition of 10 equiv. of hydrogen sulfide by building a correlation between the absorbance signal at 550 nm and the corresponding time, and the results showed that the reaction was completed within approximately 40 and 80 min of incubation, respectively (Fig. 3a). The background fluorescence of **L**_**1**_ and **L**_**2**_ was extremely weak, and within minutes, a remarkable fluorescence increase was observed, owing to the reaction of the probes with hydrogen sulfide. The pseudo-first-order rate, *k*_obs_, was found to be 2.47 × 10^−3^ and 1.21 × 10^−3^ s^−1^ for **L**_**1**_ and **L**_**2**_, respectively, by fitting the data with a single exponential function. These results revealed that the turn-on response intensity of **L**_**1**_ reached a steady state after approximately 80 min of incubation, whereas the intensity of **L**_**2**_ reached a steady state after approximately 40 min of incubation, showing an approximately 2-fold reaction rate difference between the probes. The time-dependent fluorescent response demonstrated that the probes can detect hydrogen sulfide both qualitatively and quantitatively. Specifically, the comparative solubility by substitution of a hydrophilic alkyl chain on a naphthalimide scaffold extended the reaction time for **L**_**1**_. Thus, the time scale enables these probes to detect hydrogen sulfide in real-time fluorescent imaging in living cells. We further examined the fluorescence signal change of probes with various concentrations of hydrogen sulfide. As expected, a strong emission peak at 550 nm was detected when the reaction mixture was excited at 435 nm.

Corresponding to the concentration-dependent increase, the dynamic simulation of the fluorescence response for **L**_**1**_ and **L**_**2**_ versus the NaHS concentration at approximately 550 nm was saturated at approximately 200 μM NaHS, thereby demonstrating the ability of each probe to quantify different hydrogen sulfide concentrations. When different concentrations of NaHS were added to the test solution, the fluorescence intensity increased linearly with the NaHS concentration from 10 to 200 μM (Fig. 2b). Both probes reacted with hydrogen sulfide quantitatively, even in aqueous solution. A linear function allows easy and exact analysis, and there was good linearity between the triggered fluorescence and the concentrations of hydrogen sulfide in the range of 0 to 200 μM with a detection limit of <0.3 μM (Fig. 3b). Although the total brightness of **L**_**1**_ was higher than that of **L**_**2**_, the linearity studies suggested that **L**_**1**_ and **L**_**2**_ can be used for the determination of sulfide concentrations in a biological sample. Both of the detection limits were below the previously reported range of hydrogen sulfide concentrations (20–100 μM) found in mammalian blood^10^,^11^,^14^,^49^.

**Figure 4:**
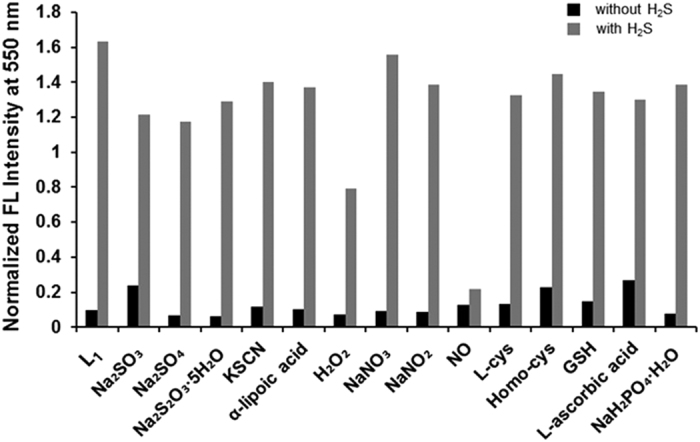
Fluorescence responses of **L**_**1**_ (10 μM) toward sulfur-containing inorganic ions (S_2_O_3_^−^, SO_4_^2−^, SO_3_^2−^, SCN^−^, 1 mM), inorganic salt (NaH_2_PO_4_, 1 mM), organosulfur compound (α-lipoic acid), reactive oxygen species (H_2_O_2_, 1 mM), reactive nitrogen species (NO, NO_3_, NO_2_, 1 mM), thiols (L-cys, homo-cys, glutathione 1 mM), L-ascorbic acid (1 mM) and NaHS (100 μM) in PBS buffer (pH 7.4) at 37 °C for 60 min. Excitation at 435 nm.

**Figure 5:**
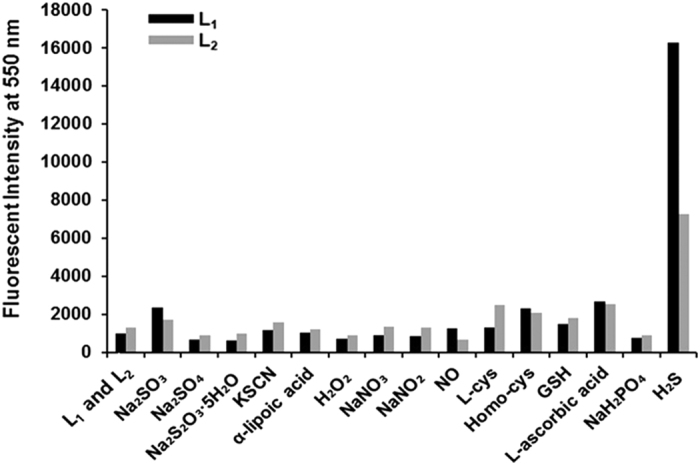
Fluorescence responses of **L**_**1**_ and **L**_**2**_ (10 μM) toward sulfur-containing inorganic ions (S_2_O_3_^−^, SO_4_^2−^, SO_3_^2−^, SCN^−^, 1 mM), inorganic salt (NaH_2_PO_4_, 1 mM), organosulfur compound (α-lipoic acid), reactive oxygen species (H_2_O_2_, 1 mM), reactive nitrogen species (NO, NO_3_, NO_2_, 1 mM), thiols (L-cys, homo-cys, glutathione 1 mM), L-ascorbic acid (1 mM) and NaHS (100 μM) in PBS buffer (pH 7.4) at 37 °C for 60 min. Excitation at 435 nm.

After establishing the time- and concentration-dependent reactivity for **L**_**1**_ and **L**_**2**_ with hydrogen sulfide, the selectivity profile of the probes was determined for hydrogen sulfide toward various biologically relevant species, such as sulfur, oxygen, and nitrogen species (RSONS). We investigated the fluorescence response by hydrogen sulfide for **L**_**1**_ only and for the mixed solution of **L**_**1**_ and analytes. Sulfur-containing inorganic ions (S_2_O^3−^, SO_4_^2−^, SO_3_^2−^, SCN^−^), an inorganic salt (NaH_2_PO_4_), an organosulfur compound (α-lipoic acid), reactive oxygen species (H_2_O_2_), a reactive nitrogen species (NO, NO_3_, NO_2_), thiols (L-cys, Homo-cys, Glutathione) and L-ascorbic acid were used as analytes and proved to be chemically inert toward the probes. Based on the previous reports using hydrogen sulfide as a reductant for azide^50^, we expected that **L**_**1**_ would have a high selectivity for hydrogen sulfide over RSONS, including biologically relevant thiols. As shown in Fig. 4 (black bar), insignificant fluorescence changes were observed from the mixed solution with analytes without hydrogen sulfide. No reaction occurred between the probe and analytes. Pronounced fluorescence changes were observed from all of the solutions in the presence of 10 equiv. (100 μM) hydrogen sulfide, indicating the excellent selectivity of the hydrogen sulfide-mediated azide-reduction mechanism (gray bar in Fig. 4). Based on the strong hydrogen sulfide sensing-properties of **L**_**1**_, another selectivity test of the fluorescence response was conducted by comparing **L**_**1**_ and **L**_**2**_ on the basis of fluorescence titration for various analytes. As expected, the fluorescent properties demonstrated the remarkable selectivity of both probes for hydrogen sulfide over the biologically relevant species; noticeable responses were not observed from other anions (Fig. 5). Therefore, the results demonstrate that the probes have a high selectivity for hydrogen sulfide, indicating their potential utility in various biological samples. Additionally, the fluorescence response intensity of **L**_**1**_ with hydrogen sulfide is relatively higher than that of **L**_**2**_, exhibiting a 2.3-fold preferential reactivity. This improvement might be attributed to the structural features, such as their structural rigidity, leading to the fluorescence intensity changes^51^. For example, the low solubility of the naphthalimide, intermolecular interactions were found to quench the fluorescence due to the formation of excimers.

**Figure 6:**
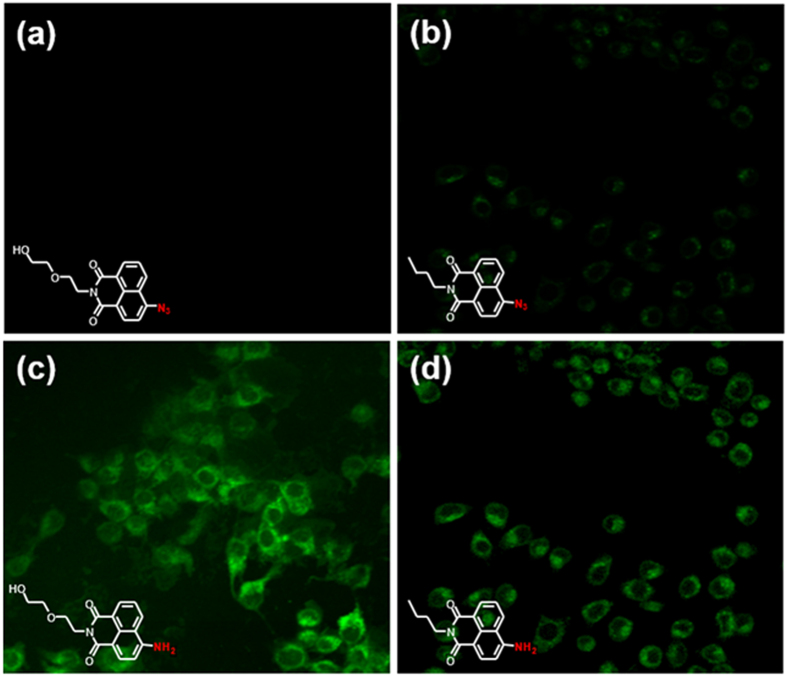
Fluorescence images of exogenous hydrogen sulfide in living cells incubated with L_1_ and L_2_. RAW264.7 cells were incubated with only 5 μM probes for 30 min (**a**,**b**) and with probes for 30 min and then 200 μM NaHS for 5 min (**c**,**d**), under 488 nm excitation. Images were acquired with emission channels of 505–605 nm (green).

To establish the potential efficacy for biological applications based on the excellent hydrogen sulfide-sensing properties of the probes, we attempted fluorescence imaging for detecting hydrogen sulfide in living cells using a confocal microscope. CCK-8 assays were conducted, and the results showed that >90% RAW264.7 cells survived after 12 h (5–20 μM incubation), and after 24 h, the cell viability remained at approximately 90%, demonstrating that both probes were minimally cytotoxic toward cultured cell lines (Fig. S10). The cell permeability of **L**_**1**_ was investigated by incubating with 5 μM **L**_**1**_ for 30 min, no fluorescence was observed (Fig. 6a). Then, the cells were incubated with 50 μM NaHS and after 5 min, they displayed green emission collected from the green channel (505–605 nm), establishing the efficacy of **L**_**1**_ for detecting endogenously produced hydrogen sulfide in cells. Because the high selectivity and sensitivity of **L**_**1**_ have been demonstrated for hydrogen sulfide *in vitro*, we examined the ability of **L**_**1**_ to detect changes in the hydrogen sulfide levels in living cells by using a RAW264.7 cell model. Fluorescence images of hydrogen sulfide in RAW264.7 cells incubated with 5 μM **L**_**1**_ and **L**_**2**_ for 30 min and 200 μM NaHS for additional 5 min were observed, displaying enhanced green fluorescence response, respectively (Fig. 6c,d). Interestingly, along with the various fluorescent spectroscopic results, a marked difference in fluorescence intensity was also observed, showing a stronger fluorescent response of **L**_**1**_. **L**_**1**_ provided a higher turn-on response compared to **L**_**2**_ for the detection of hydrogen sulfide in living cells, which might be due to the increased hydrophilicity and cellular retention of **L**_**1**_ relative to **L**_**2**_.

Incubation of RAW264.7 cells with **L**_**1**_ (5 μM) for 1 h at 37 °C was followed by the addition of different concentrations of NaHS (50, 100, 150 and 200 μM) and then incubation for another 1 h. After removing the excess NaHS, the cells were subsequently imaged using a confocal fluorescence microscope.

**Figure 7:**
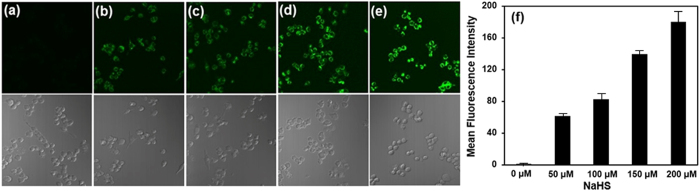
Fluorescence imaging of exogenous sulfide in living RAW264.7 cells with L_1_ upon excitation at 488 nm. Cells were incubated with 5 μM **L**_**1**_ for 1 h. (**a**) **L**_**1**_ (5 μM) without NaHS as negative control (**b**) **L**_**1**_ (5 μM) with 50 μM NaHS (**c**) **L**_**1**_ (5 μM) with 100 μM NaHS (**d**) **L**_**1**_ (5 μM) with 150 μM NaHS (**e**) **L**_**1**_ (5 μM) with 200 μM NaHS. (**f**) The cell body regions in the visual field were selected (n = 10) as the regions of interest (ROI).

As shown in Fig. 7, RAW264.7 cells treated with only **L**_**1**_ as a control showed no fluorescence, at 505–605 nm under excitation of 488 nm. However, in the presence of **L**_**1**_ and NaHS, RAW264.7 cells showed strong fluorescence at only 50 μM NaHS. The fluorescence intensity increased with increases in the NaHS concentration. These results demonstrate that **L**_**1**_ has potential in visualizing hydrogen sulfide in living cells, which can likely be extended to assays involving biological fluids such as serum, blood, or tissue homogenates. Also, the availability of this water-soluble fluorescent probe will significantly help the effort of making biocompatible fluorescent sensors for the detection of hydrogen sulfide in living cells.

**Figure 8:**
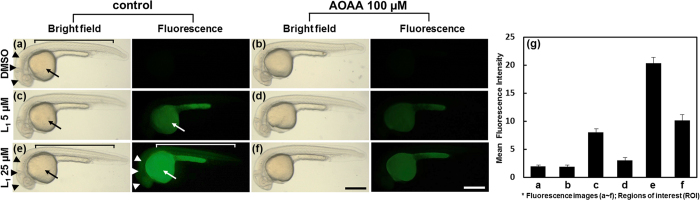
Detection of endogenous hydrogen sulfide by **L**_**1**_ in zebrafish embryos *in vivo*. All embryos at 27 h postfertilization. Zebrafish embryos were imaged after 2 h pretreatment with control (deionized water, left column (**a**,**c**,**e**)) or 100 μM AOAA pretreatment (right column (**b**,**d**,**f**)), followed by incubation of embryos with 0.01% DMSO control (**a**,**b**), 5 μM **L**_**1**_ (**c**,**d**), 25 μM **L**_**1**_ (**e**,**f**) for 30 min. (**g**) The measurement of the mean fluorescence intensity of the whole embryonic body as region of interest (ROI). Arrow: yolk; arrowheads: brain; bracket: trunk. Scale bar = 400 μm.

To further establish **L**_**1**_ as an *in vivo* hydrogen sulfide reporter, we next examined its endogenous detection using zebrafish embryos. By taking advantage of their transparency, we treated **L**_**1**_ into the developing zebrafish embryos at 24 h postfertilization. Incubation of 5 μM **L**_**1**_ with zebrafish embryos elicited fluorescent signals mainly in the yolk (arrows in Fig. 8c). Incubation of 25 μM **L**_**1**_ produced strong signals in the brain and the spinal cord (arrowheads and bracket in Fig. 8e, respectively) as well as in the yolk, suggesting that **L**_**1**_ can effectively detect endogenously produced hydrogen sulfide. In order to validate the specificity of **L**_**1**_ against hydrogen sulfide, we pretreated zebrafish embryos for 2 h with aminooxyacetic acid (AOAA), a frequently used inhibitor against cystathionine-*β*-synthase (CBS), a key enzyme for hydrogen sulfide synthesis^52^, followed by **L**_**1**_ incubation (Fig. 8b,d,f). Upon AOAA pretreatment, the fluorescence intensity in the yolk, brain, and the trunk detected by **L**_**1**_ dramatically decreased up to less than 50% (Fig. 8d,f, compared to 8c, 8e, respectively; Fig. 8g), corroborating the finding that **L**_**1**_ detects endogenously produced hydrogen sulfide. In addition, **L**_**1**_ appears not to be toxic to embryos with a range of doses (5~25 μM) that were tested since no obvious deformity or survivability were found upon treatment (Fig. 8a,c,e, and data not shown).

## Conclusions

In conclusion, a novel naphthalimide-based reduction-sensitive fluorescence sensor was developed for hydrogen sulfide detection in aqueous solutions, including in living cells. The probes, **L**_**1**_ and **L**_**2**_, are simple in structure, easy to synthesize, stable, and amenable to long-term storage. **L**_**1**_ was highly selective for sulfide among 14 anions tested and other common reducing species, with a detection limit of <0.3 μM in PBS buffer solution without the use of an organic co-solvent. The fluorescence enhancement of **L**_**1**_ upon hydrogen sulfide treatment reached more than 70-fold, and the quantum yield of **L**_**1**_ after hydrogen sulfide treatment was 0.72. In addition, **L**_**1**_, compared to **L**_**2**_ had a two-fold faster reaction rate toward hydrogen sulfide and better stability through the enhanced solubility in PBS buffer. The time-dependent fluorescent response demonstrated that probes could detect hydrogen sulfide both qualitatively and quantitatively. The obtained linear relationship for the concentration covered the reported endogenous concentration range of hydrogen sulfide. **L**_**1**_ provided a higher turn-on response compared to that of **L**_**2**_ for the detection of hydrogen sulfide in living cells, thus demonstrating the potential for visualizing hydrogen sulfide in living cells and zebrafish embryos *in vivo*, which can likely be extended to assays involving biological fluids, such as serum, blood, or tissue homogenates. We are actively seeking more sensitive and responsive methods for the fluorescence imaging of hydrogen sulfide in living cells, tissues, and animals, as well as the utilization of these probes to study the endogenous production of hydrogen sulfide in living cells and its contributions to physiological and pathological processes.

## Methods

### Materials

6-Azido-2-(2-(2-(2-hydroxyethoxy)ethoxy)ethyl)-1H-benzo[de]isoquinoline-1,3 (2H)-dione (**L**_**1**_) was synthesized in our laboratory. 4-Bromo-1,8-naphthalic anhydride was purchased from TCI (Tokyo, Japan). 2-[2-(2-Aminoethoxy)ethoxy]ethanol and sodium azide were purchased from Sigma-Aldrich. A mouse leukemic monocyte macrophage cell line (RAW264.7) was obtained from the cell bank of the ATCC. **L**_**1**_ (6.0 mM, 2.5 mL) was prepared in dimethyl sulfoxide (DMSO) and stored at −18 °C in the dark. All other reagents and chemicals were from commercial sources, were of analytical reagent grade, and were used without further purification. The progress of the reactions was monitored by TLC on precoated Merck silica gel plates (60 F_254_).

### Instruments

^1^H-NMR and ^13^C-NMR spectra for the structural analyses of the probes were obtained with Varian Inova 400NB or Inova 600NB spectrometers. UV/Vis and fluorescence spectra were obtained with a Beckman Coulter DU800 spectrophotometer and Scinco Fluoromate FS-2 spectrometer, respectively.

### Spectroscopic Measurements

Spectroscopic measurements were performed in PBS (10 mM, pH 7.4) buffer at 37 °C. Stock solution of **L**_**1**_ and **L**_**2**_ were dissolved into DMSO with a concentration of 6.0 mM and 7.8 mM, and stored at −20 °C until immediately before use. A volumetric flask was charged with 50 mL of PBS buffer. After injection of **L**_**1**_ (41 μL) and **L**_**2**_ (32 μL) stock solution via micropipette, the UV-vis absorption (λ_abs_ = 340–400 nm and 400–500 nm) and fluorescence spectra (λ_ex_ = 435 nm, λ_em_ = 500–700 nm) was recorded. Aqueous stock solutions of NaHS, L-cysteine, homocysteine, glutathione, L-ascorbic acid, α-lipoic acid, NaS_2_O_3_, Na_2_SO_3_, Na_2_SO_4_, SCN^−^, NaH_2_PO_4,_ NO, NO_2_, NO_3_ and H_2_O_2_ was then injected via micropipette. The reaction cuvettes were incubated at 37 °C during the experiment.

### Determination of Detection Limit

The fluorescence of seven blank cuvettes containing **L**_**1**_ (5 μM, λ_ex_ = 435 nm, λ_em_ = 500–700 nm) was recorded after incubation at 37 °C in PBS buffer (10 mM, pH 7.4). Then **L**_**1**_ was treated with NaHS at various concentrations (10, 30, 50, 100, 150, and 200 μM), and the fluorescence spectra were measured after incubation for 90 min at 37 °C. Each data point represents at least three trials. A linear regression was constructed using the background-corrected fluorescence measurements, and the detection limit was determined to be concentration at which the fluorescence equals that of [blank + 3σ]. The detection limit was calculated with the following equation: Detection limit = 3 σ/*k*, *k* = the slop of emission intensity versus NaHS concentration graph, σ = the standard deviation of 7 blank measures.

### Determination of the fluorescence quantum yield

Fluorescence quantum yields for **L**_**1**_ were determined by using Rhodamine 6G (Φ_F_ = 0.95 in ethanol) as a fluorescence standard. The quantum yield was calculated using the following equation:




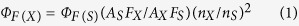




where Φ_F_ is the fluorescence quantum yield, A is the absorbance at the excitation wavelength, F is the area under the corrected emission curve, and n is the refractive index of the solvents used. Subscripts S and X refer to the standard and to the unknown, respectively.

### Synthesis of product 1

4-Bromo-1,8-naphthalic anhydride (0.4643 g, 1.6757 mmol) was dissolved in ethanol (9.3 mL) and 2-[2-(2-aminoethoxy)ethoxy]ethanol (0.250 g, 1.6757 mmol) was added and stirred in the refluxing ethanol at 80 °C for 2 h. TLC showed the consumption of starting materials at this stage. The reaction mixture was cooled, and the solvent was evaporated. The product was purified by column chromatography on silica gel using ethyl acetate/hexane (3:1) as the eluent. Product **1** was achieved as a light yellow solid (569.5 mg, 83% yield). ^1^H-NMR (CDCl_3_, 600 MHz): δ 8.56 (d, 1H, *J* = 7.2 Hz), 8.43 (d, 1H, *J* = 9.0 Hz), 8.31 (d, 1H, *J* = 7.8 Hz), 7.95 (t, 1H, *J* = 7.8Hz), 7.78 (t, 1H, *J* = 16.2 Hz), 4.42 (t, 1H, *J* = 12Hz), 3.86 (t, 1H, *J* = 12.6 Hz). ^13^C-NMR (CDCl_3_, 600 MHz): δ 163.52, 163.49, 133.14, 131.98, 131.15, 130.98, 130.29, 130.22, 128.70, 127.97, 122.71, 121.86, 72.48, 70.39, 70.04, 67.91, 61.64, 39.20. HRMS (*m/z*); Calcd for [M + Na]^+^ 430.0261, found 430.0268.

### Synthesis of L_1_

0.5695 g (1.3949 mmol) of product **1** was dissolved in 11.6 mL of dry DMF, and NaN_3_ (0.9069 g, 13.949 mmol) was added. After stirring the mixture for 6 h at 80 °C while monitoring TLC, the solution was diluted with H_2_O and extracted with ethyl acetate. The organic layer was separated and dried over Na_2_SO_4_. The product was concentrated in vacuo, affording **L**_**1**_ as a yellow solid (419 mg, 81% yield). ^1^H-NMR (CDCl_3_, 600 MHz): δ 8.51 (d, 1H, *J* = 7.2 Hz), 8.43 (d, 1H, *J* = 7.8 Hz), 8.29 (d, 1H, *J* = 7.8 Hz), 7.66 (t, 1H, *J* = 15.6Hz), 7.35 (d, 1H, *J* = 7.8 Hz), 4.40 (t, 1H, *J* = 12.6 Hz), 3.85 (t, 1H, *J* = 12.6 Hz). ^13^C-NMR (CDCl_3_, 600 MHz): δ 163.81, 163.36, 143.25, 132.05, 131.57, 128.80, 126.68, 123.96, 122.18, 118.43, 114.51, 77.10, 72.45, 70.38, 70.00, 67.92, 61.61, 39.00. HRMS (*m/z*); Calcd for [M + Na]^+^ 393.1169, found 393.1170.

### Synthesis of product 2

4-Bromo-1,8-naphthalic anhydride (4.0 g, 14.43 mmol) was dissolved in ethanol (80 mL), and butylamine (1.005 g, 14.43 mmol) was added. The reaction mixture was stirred in the refluxing ethanol for 24 h. After cooling to room temperature, the mixture was filtered and dried under reduced pressure to yield 4.60 g (96%) of a yellow solid. ^1^H-NMR (CDCl_3_, 400 MHz): δ 8.66 (dd, 1H, *J* = 6.8, 0.8 Hz), 8.57 (dd, 1H, *J* = 8.4, 0.8 Hz), 8.42(d, 1H, *J* = 7.6 Hz), 8.05 (d, 1H, *J* = 7.6Hz), 7.86 (t, 1H, *J* = 15.6, 7.2 Hz), ^13^C-NMR (CDCl_3_, 400 MHz): δ 163.60, 133.16, 131.96, 131.15, 131.06, 130.61, 130.13, 128.04, 123.16, 122.30, 76.98, 40.35, 30.14, 20.37, 13.79. HRMS (*m/z*); Calcd for [M + Na]^+^ 354.0, found 354.02.

### Synthesis of L_2_

2 g (6.02 mmol) of product **2** was dissolved in 50 mL of dry DMF, and NaN_3_ (3.91 g, 60.20 mmol) was added. After stirring the mixture for 6 h at 80 °C, the solution was diluted with H_2_O and extracted with EtOAc. The organic phase was washed with H_2_O and brine, dried over Na_2_SO_4_, and concentrated in vacuo. The residue was placed at room temperature overnight, and then, ether was added (to the residue yellow solid and triturated at room temperature) The yellow solid was filtered off and dried in vacuo to give **L**_**2**_ (1.0717 g, yield: 60.49%). ^1^H-NMR (CDCl_3_, 400 MHz): δ 8.63 (dd, 1H, *J* = 7.6, 1.2 Hz), 8.58 (d, 1H, *J* = 8.0 Hz), 8.43 (dd, 1H, *J* = 8.8, 1.2 Hz), 7.75 (t, 1H, *J* = 15.6, 7.2Hz), 7.47 (d, 1H, *J* = 8 Hz), ^13^C-NMR (CDCl_3_, 400 MHz): δ 163.95, 163.53, 143.32, 132.12, 131.61, 129.12, 126.81, 124.32, 122.67, 122.67, 118.97, 114.62, 76.98, 40.24, 30.19, 20.35, 13.80. HRMS (*m/z*); Calcd for [M + Na]^+^ 317.1, found 317.1.

### RAW264.7 murine macrophages culture and imaging using L_1_

RAW264.7 murine macrophages were cultured in Dulbecco’s Modified Eagle’s Medium (DMEM) supplemented with 10% fetal bovine serum (FBS) in an atmosphere of 5% CO_2_ and 95% air at 37 °C. After 24 h, the cover slips were rinsed 3 times with Dulbecco’s Phosphate Buffered Saline (DPBS) to remove the media and were then cultured in DPBS for later use. For the verification procedure, 5 μM of **L**_**1**_ was added to the above cellular samples and incubated for 30 min. Then, the samples were rinsed 3 times with DPBS. The cells were incubated with NaHS (0, 50, 100, 150 and 200 μM) in the medium for 60 min. Prior to imaging, the cells were washed 3 times with DPBS, and the fluorescence images were acquired on a confocal microscope (Olympus Fluoview 1000) using an oil-immersion 60× objective.

### Cytotoxicity test (CCK-8 assays)

HeLa cells were plated in flat-bottomed, 96-well plates at a the density of 5,000 cells/well in 200 μL of DMEM (GIBCO, 11885) supplemented with 10% (v/v) FBS and 1% penicillin/streptomycin in a humidified incubator in 5% CO_2_ in air at 37 °C. Following incubation for 24 h, **L**_**1**_ and **L**_**2**_ (5% DMSO as a co-solvent for only **L**_**2**_) were added to the above cellular samples plates. After incubation for 30 min, 10 μL of CCK-8 solution (Dojindo, Japan) was added to each plate well, and the cells were further incubated for 30 min. The absorbance at 450 nm was measured with a microplate reader (SpectraMax M2 / Molecular devices).

### Preparation and imaging of chemical-treated zebrafish embryos

Wild type adult zebrafish (AB line) reared at 28 °C with the light cycle of 14 h light/10 h dark were group-mated. Spawned eggs were staged according to Kimmel *et al.*^53^. Embryos at 24 h postfertilization were pretreated with O-(Carboxymethyl) hydroxylamine hemihydrochloride (AOAA, Sigma, Cat. #C13408) 100 μM for 2 h in E3 egg water at 28.5 °C incubator, followed by three time (5 min each) washes with E3 egg water. After washes, embryos were transferred to **L**_**1**_ solution with two concentrations of 5 μM or 25 μM for 30 min in E3 egg water at room temperature, again with three time (5 min each) washes with E3 egg water afterwards. The embryos were embedded alive in the 2.5% methyl cellulose, and fluorescence signals were visualized under the Olympus SZX16 stereo microscope equipped with the excitation filter GFP-A illuminated using a mercury lamp (Olympus, U-RFL-T). Images were captured using Olympus XC10 camera. All zebrafish husbandry and animal care were carried out in accordance with guidelines from the Korea Research Institute of Bioscience and Biotechnology (KRIBB) and all experimental protocols were approved by KRIBB-IACUC (approval number: KRIBB-AEC-16036).

## Additional Information

**How to cite this article**: Choi, S.-A. *et al.* Structural effects of naphthalimide-based fluorescent sensor for hydrogen sulfide and imaging in live zebrafish. *Sci. Rep.* **6**, 26203; doi: 10.1038/srep26203 (2016).

## Supplementary Material

Supplementary Information

## Figures and Tables

**Figure 1:**
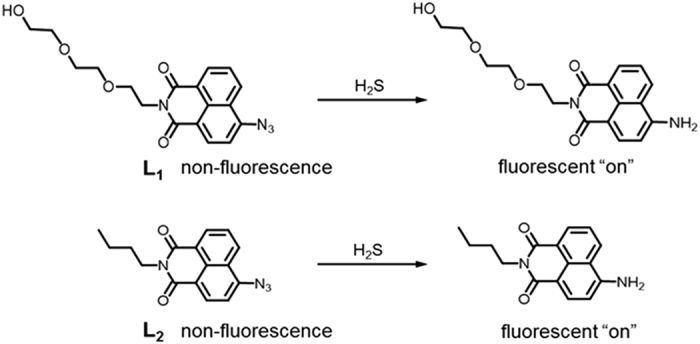
L_1_ and L_2_ as fluorescent probes for hydrogen sulfide.
