# Supplementary material for: Structural effects of naphthalimide-based fluorescent sensor for hydrogen sulfide and imaging in live zebrafish
Source: Sci Rep. 2016 May 18;6:26203. doi: 10.1038/srep26203 (PMC4870630; doi:10.1038/srep26203)
Supplement: Supplementary Information [file srep26203-s1.pdf]

Supplementary Information for

Structural effects of naphthalimide-based fluorescent sensor for hydrogen sulfide and imaging in live zebrafish

Seon-Ae Choi[a,b], Chul Soon Park[a], Oh Seok Kwon[a], Hoi-Khoanh Giong[a], Jeong-Soo Lee[a,c], Tai Hwan Ha[a,b,*] and Chang Soo Lee[a,b,*]

[a]*BioNanotechnology Research Center, Korea Research Institute of Bioscience and Biotechnology (KRIBB), 125 Gwahak-ro Yuseong-gu, Daejeon 305-806, South Korea*

[b]*Nanobiotechnology (Major), University of Science & Technology (UST), 125 Gwahak-ro Yuseong-gu, Daejeon 305-806, South Korea*

[c]*Functional Genomics (Major), University of Science & Technology (UST), 125 Gwahak-ro Yuseong-gu, Daejeon 305-806, South Korea*

*Co-corresponding authors; E-mail: cslee@kribb.re.kr & taihwan@kribb.re.kr

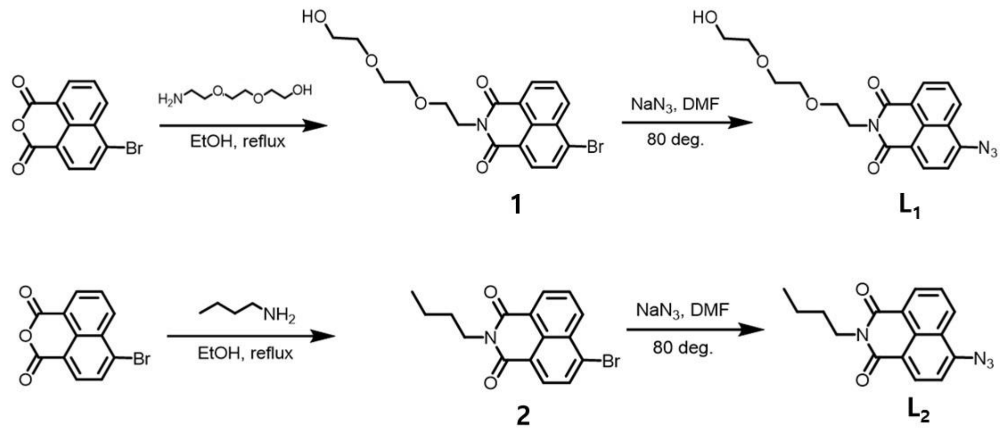

Figure S1. Synthesis of **L1** and **L2**.

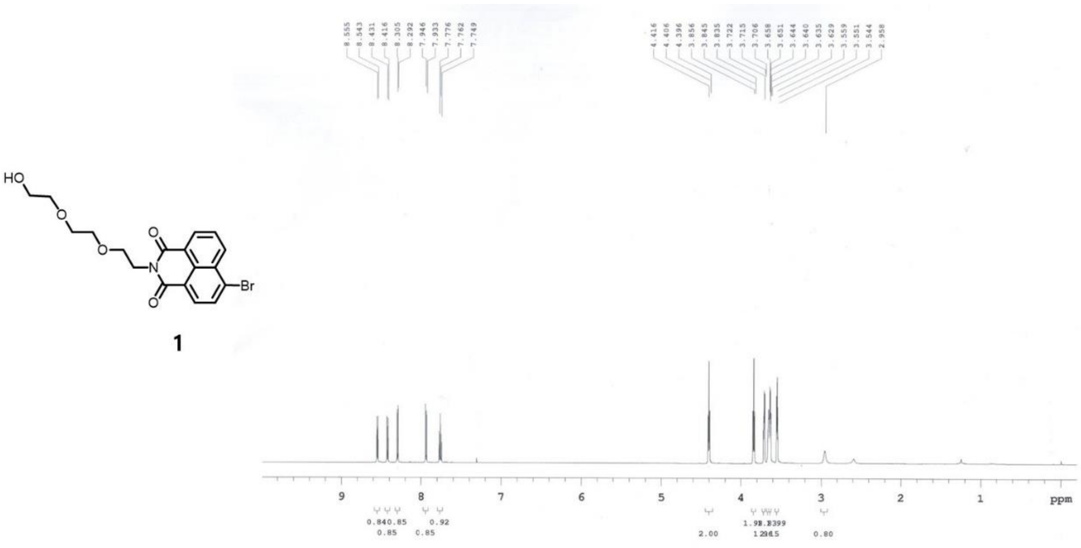

Figure S2. ^{1}H NMR spectrum of product **1** in $CDCl_3$.

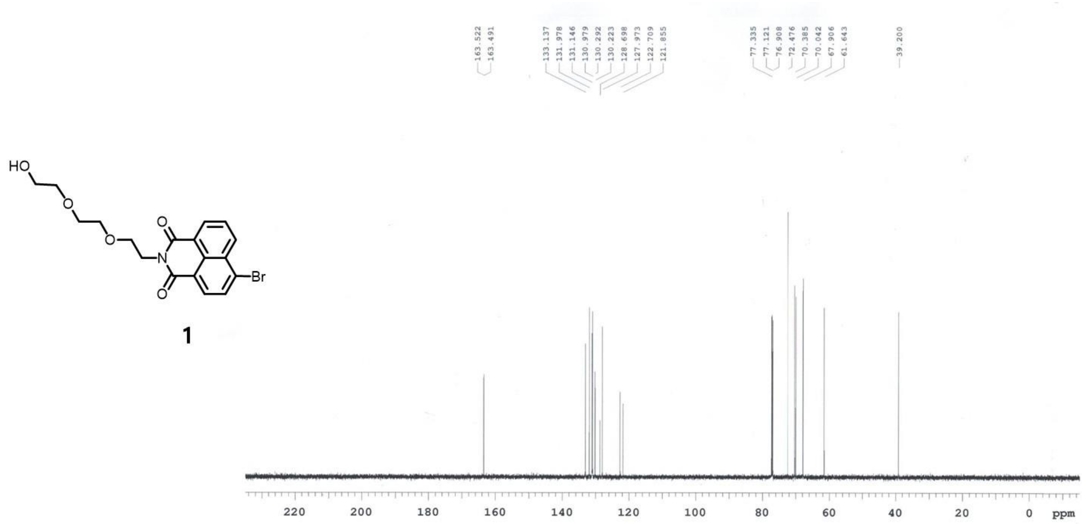

Figure S3. ^{13}C NMR spectrum of product **1** in $CDCl_3$.

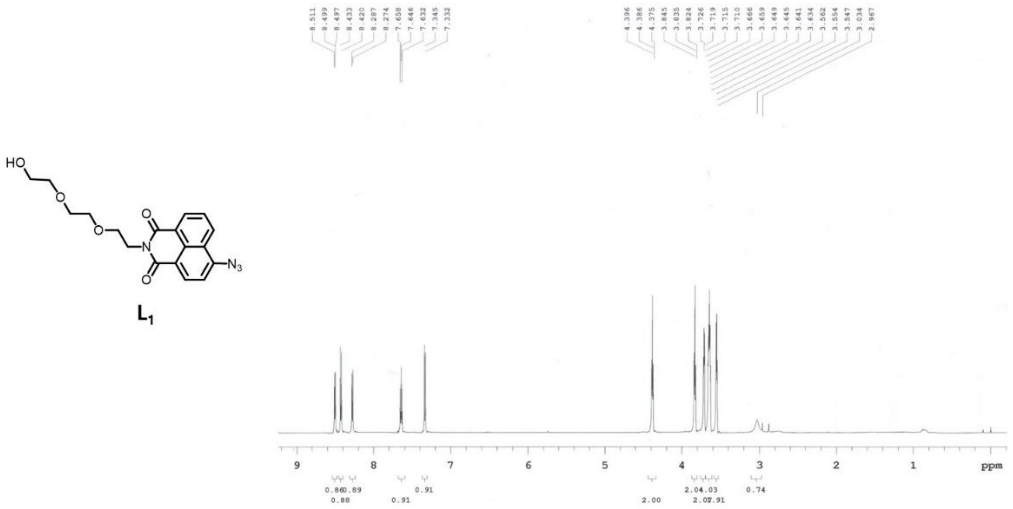

Figure S4. ^{1}H NMR spectrum of $\mathbf{L_1}$ in $CDCl_3$.

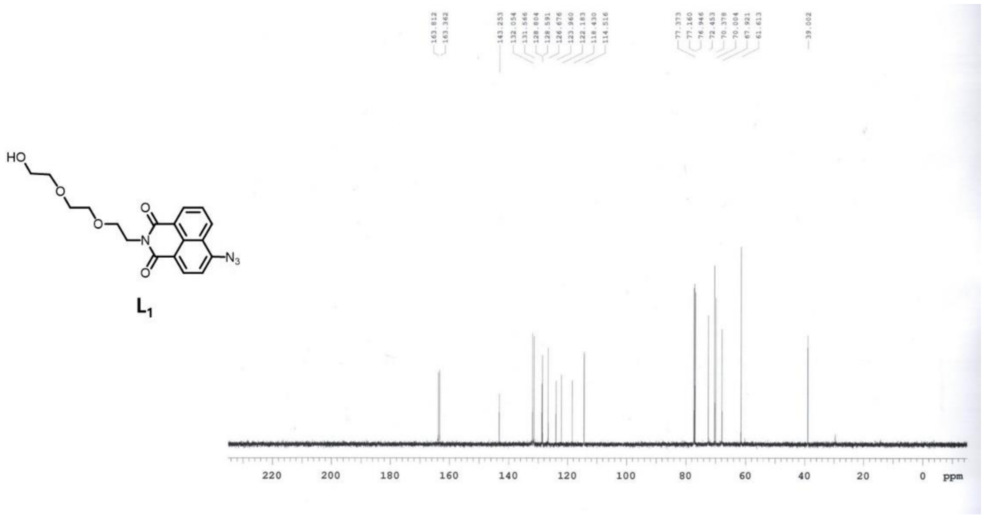

Figure S5. ^{13}C NMR spectrum of **L_1** in $CDCl_3$.

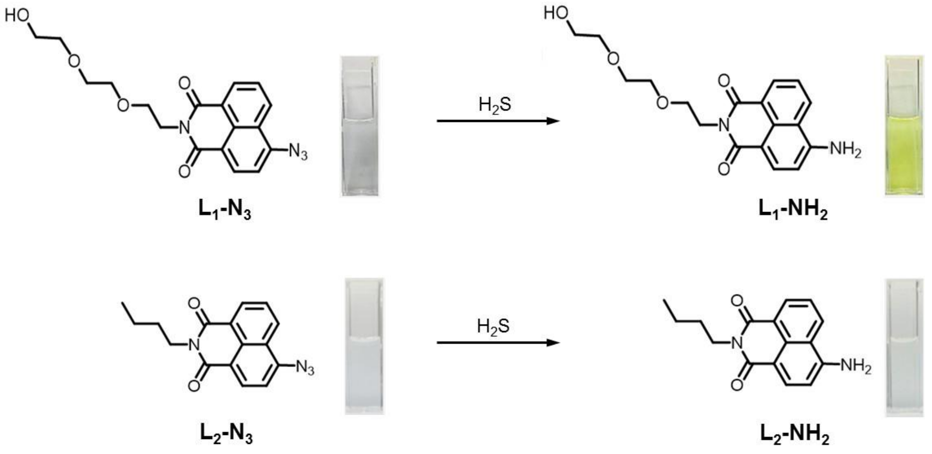

Figure S6. Color change of solution of **L_1** and **L_2** of with the addition of hydrogen sulfide.

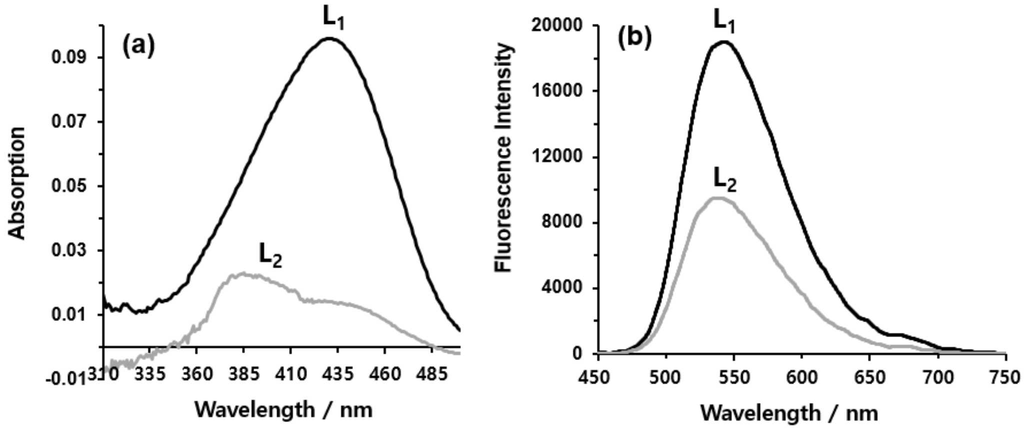

Figure S7. UV–vis absorption and fluorescence spectra of **L1** (10 μM) and **L2** (10 μM) in the presence of hydrogen sulfide (200 μM) in aqueous solution.

We added a data about solubility test using UV-vis and fluorescence spectroscopy and conducted the additional solubility test for the probes (**L1** and **L2**), controlling the concentration of DMSO (0~10 %) in PBS. First, **L1** clearly showed a little difference in fluorescent intensity before and after reaction with hydrogen sulfide, compared to the fluorescent intensity between with no DMSO and with 10 % DMSO. It means that **L1** is almost soluble in aqueous solution regardless of mixing organic solvent. Moreover, comparing to the fluorescent intensity of **L2**, which increase as to increase of the concentration of DMSO, **L1** showed much larger fluorescent intensity than **L2** by excellent solubility.

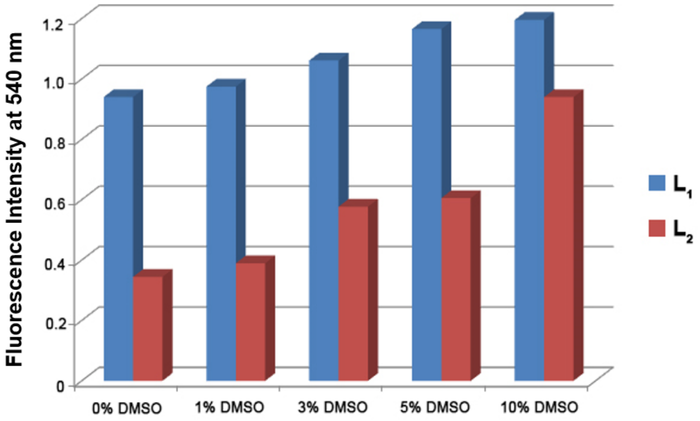

Figure S8. Fluorescence spectra of 10 μM **L1** (a) and **L2** (b) with NaSH (200 μM) in PBS/DMSO (0~10 %) at 37℃ for 30 min. Excitation at 435 nm (Ex Slit: 2.5 nm, Em Slit: 5 nm).

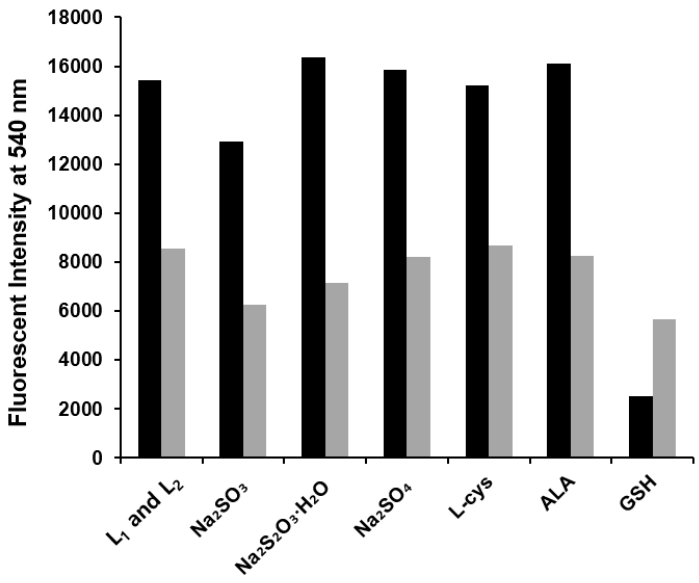

Figure S9. Fluorescence responses of **L1** (10 μM) and **L2** (10 μM) towards NaHS (200 μM) and sulfur containing inorganic ions ($S_2O_3^-$,SO_4^{2-}, SO_3^{2-} 1 mM), and thiols (Cys 1 mM, ALA 1 mM, GSH 1 mM) in PBS buffer (pH=7.4) at 37°C for 60 min. Excitation at 435nm.

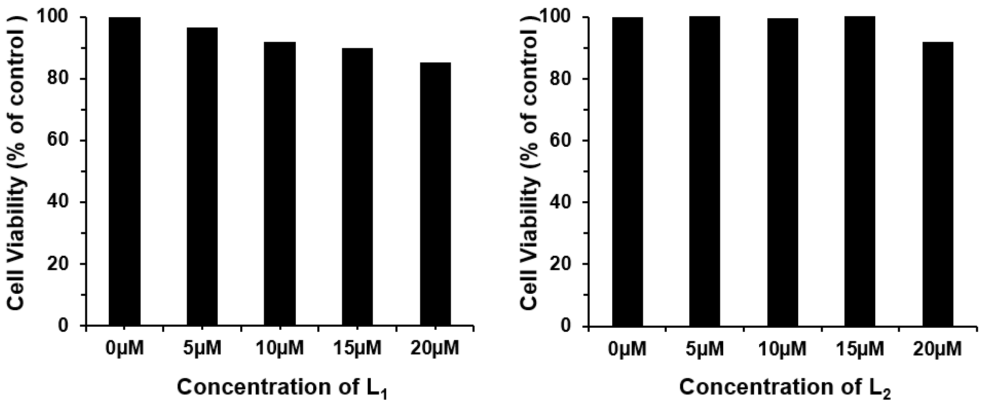

Figure S10. CCK-8 assays of RAW264.7 cells in the presence of different concentrations of **L1** and **L2**
